# Immunotherapy for Chronic Hepatitis B using HBsAg-based Vaccine Formulations: From Preventive Commercial Vaccines to Therapeutic Approach Julio Cesar Aguilar,

**DOI:** 10.5005/jp-journals-10018-1109

**Published:** 2014-07-28

**Authors:** Julio Cesar Aguilar, Lobaina Y

**Affiliations:** 1 Department of Hepatitis B, Biomedical Research Unit, Center for Genetic Engineering and Biotechnology, Havana, Cuba

**Keywords:** HBV, Preventive vaccine, Chronic hepatitis B, Therapy, Immune therapy.

## Abstract

Despite the existence of effective prophylactic vaccines, hepatitis B virus (HBV) infections remain a major public health problem. It has been estimated that about 370 million people are chronically infected with this virus worldwide. These individuals act as a reservoir for viral spread and chronic infection also increases the risk of liver diseases, such as cirrhosis and hepatocellular carcinoma. Current antiviral therapies fail to control viral replication in the long term in most patients. Viral persistence has been associated with a defect in the development of HBV-specific cellular immunity. The limitations of the current available therapies underline the need for alternative therapies. Specific immunotherapeutic strategies target not only the induction or stimulation of CD4(+) and CD8(+) T-cell responses but also the induction of proinflammatory cytokines capable of controlling viral replication. Therapeutic vaccination has been extensively studied in chronic hepatitis B (CHB) based in the properties of hepatitis B surface antigen (HBsAg) and taking advantage of its previous use in preventive vaccination. In this sense, pioneer studies were carried out employing HBsAg-based vaccines, including prophylactic commercial vaccines and HBsAg-based formulations with novel adjuvants. The results and general knowledge coming from these studies are discussed in the present review. The decision on developing new generations of vaccines including new antigens or formulations should take into account the experience with HBsAg-based vaccine formulations in order to decide about changing the vaccine antigen or adding new antigens to improve the composition.

**How to cite this article:** Aguilar JC, Lobaina Y. Immunotherapy for Chronic Hepatitis B using HBsAg-based Vaccine Formulations: From Preventive Commercial Vaccines to Therapeutic Approach. Euroasian J Hepato-Gastroenterol 2014;4(2):92-97.

## INTRODUCTION

Despite the universal vaccination of neonates and infants during the last decades and the subsequent reduction in the incidence of new infections with hepatitis B virus (HBV), chronic HBV infection remains a significant public health problem worldwide. Nowadays, more than 350 million people are persistently infected and two billion people show evidence of past or current infection. The state of chronicity correlates with an increased risk of developing liver cirrhosis, hepatocellular carcinoma and other complications like portal hypertension and liver failure. As a consequence, one million people die each year worldwide.^1^

Currently, several drugs are recommended for treatment of patients with CHB. These drugs can be divided into two main groups based on their mechanism of action, namely immunomodulatory drugs like interferon and antiviral drugs, including lamivudine, adefovir, entecavir, tenofovir and telbivudine.^2^ These drugs have a limited efficacy, rarely produce a sustained response and their prolonged use is associated to important adverse events.^3^ The use of vaccine formulations as immunotherapeutic strategies in the treatment of CHB constitutes an attractive approach. This review focuses on the use of hepatitis B surface antigen (HBsAg)-based vaccine formulations on CHB treatment and discuss the general conclusions coming from these studies and the potentialities of using HBsAg antigens in the future developments.

### Immunotherapy with Conventional Preventive Vaccines

The available therapies in the present time have not been able to control the virus in the long term in most treated patients. The viral persistence has been associated with a defect in the development of the anti-HBV cell immunity. From the beginning of the 1980s, vaccine strategies aimed at broadening and potentiate the weak T cell response of CHB patients have been disclosed.

These immunotherapeutic strategies initially used commercial anti-HBV vaccines with the objective of inducing CD4+ and CD8+ specific responses against HBV and proinflammatory cytokines able to control the viral replication.

Almost all commercial preventive vaccines were assayed alone or together with conventional antiviral therapies. In this chapter, studies of vaccines administered without other antiviral treatments are addressed as well as therapeutic vaccine studies in conditions of reduced viral load.

The immunotherapy with commercial vaccines also proposed a larger number of administrations and explored alternative parenteral routes.

Heptavax B

The first vaccination study aimed at treating CHB was published in 1982. This study used a prophylactic vaccine based on plasma-derived HBsAg (Heptavax-B). The target was to subvert the tolerance in the immune response to HBsAg, immunizing the patient with antigens differing slightly from the tolerogenic antigen.^4^

With this purpose, batches of HBsAg particles from different subtypes (adw and ayw) were treated with formalin and digestive enzymes. These particles were adjuvanted in alum hydroxide and were inoculated to 16 chronic carriers infected with the adw subtype. These patients received 6 monthly injections of HBsAg adw as well as ayw subtypes. The primary objective of the study was the induction of anti-HBs antibodies and the elimination of HBsAg.^4^

Exacerbations of the serum transaminase levels were observed in two subjects starting from the second dose. The study was stopped since the increases were attributed to non-A and non-B hepatitis infection by a potentially contaminated vaccine lot. In this way only five patients received the complete course of six doses. One subject became sero-negative to HBeAg and generated antibodies against this antigen. None of the subjects cleared HBsAg neither produced anti-HBsAg antibodies.^4^

These results, although very limited to arrive to conclusions, may be interpreted nowadays in a different way.

The objective of achieving seroconversion to the S antigen is now considered as something very improbable and the increases of the transaminase levels usually precede seroconversion, so that is not necessarily a negative event.

GenHevac and Recombivax

A pilot study using another prophylactic vaccine Gen-Hevac B (Aventis Pasteur, France) was reported by Pol et al, 10 years after the first study. This vaccine contained the HBsAg including Pre S2 region. The antigen was produced by recombinant procedures in CHO cells.^5^

A total of 32 HBsAg carriers, 29 of them HBeAg positive were included in the study. The patients received a total of three doses at one month intervals. The main objective of this study was the reduction of the viral concentration in the sera of treated patients. Six months after the first dose the virus was reduced to undetectable levels in 12 patients (37.5%) and a significant reduction in other three patients was observed. A total of 21 patients were treated subsequently with interferon for a period of 4 months and after a follow-up period the viral DNA disappeared in 18 out of 32 patients (53.1%). Anti-HBeAg antibodies developed in 15 patients, in some cases up to 2 years after the virus clearance. A transaminase increase preceded the virus clearance in 13 out of the 18 patients that cleared HBV. These results were compared with the historical data were the spontaneous elimination of HBV replication was observed in a period of 6 months only in about 2% of the patients. In this study, it was concluded that the anti-HBV vaccination substantially reduced HBV replication in about 50% of the chronic carriers. It is important to consider that, in this study, the detection limit of the viral load quantification assay was limited by its sensitivity. At that time, limits of approximately 105 DNA copies/mL were prevalent while nowadays limit has come down to 50 copies per mL for routine PCR-based tests.

Afterward, a larger multicenter placebo controlled study was carried out, to confirm the former results. In this study, a control group was additionally introduced inoculating Recombivax vaccine (Merck Sharp & Dohme-Chibret, France) containing only HBsAg, produced in yeast. Out of a total of 152 patients, three randomized groups were created that received 6 doses of GenHevac, Recombivax or placebo in the following way: three doses at monthly intervals, followed by three doses at three months intervals. All this was administered in a period of 12 months.^6^ The primary objective was, again, the reduction of the DNA levels below the detection limit. Although a significant difference was observed at 6 month (3%, 20% and 22%) for the groups inoculated with placebo, GenHevac and Recombivax respectively; this difference disappeared at 12th month, when 30%, 25% and 35% were registered respectively. Concluding, it was not possible to observe a clear clinical benefit and the pre-S2 antigen present in this vaccine did not seem to have any additional effect.

The results, apparently contradictory, have led to the evaluation of the same vaccine in other group of patients. A group of 31 inactive HBV carrier patients were vaccinated with three monthly inoculations of Genhevac and the follow-up was carried out for 12 months.^7^ Forty nontreated subjects were followed as control. Three out of the 31 vaccinated subjects cleared HBsAg, which did not occurred in none of the patients of the control group. The loss of the antigen was followed by the presence of anti-HBsAg antibodies, occurring two to three months after the start of the vaccination in all the cases.^7^ In another trial, the same therapeutic schedule was evaluated in immune-tolerant children with low or normal transaminase levels (below 1.5 times the upper normal limit). There were no significant differences regarding efficacy between the 43 vaccinated children and the 31 patients that received placebo.^8^

Hepagene (Posteriorly renamed as Hepacare)

Another prophylactic vaccine also produced in mammalian cells formerly known as Hepagene (Medeva Ltd, UK) containing all the HBsAg variants (L, S and M) was evaluated adsorbed in aluminum hydroxide. With this vaccine, it had been reported in healthy voluntaries the induction of anti-HBs antibody levels higher than those obtained with the conventional yeast vaccines^9^ as well as higher responses in nonresponder subjects to the classic vaccination.^10,11^ This vaccine also induced a strong T helper cell 1 (Th1) type cellular immune response in mice^12^ and chimpanzees.^13^

A total of 24 CHB patients were included in a pilot trial. These patients were initially positive to HBV-DNA and HBeAg. The patients received total of 8 doses of 20ug of the vaccines in two cycles of 4 inoculations, separated by 5 months from each other.^14^ At the end of the schedule, 8 out of the 22 patients that completed the schedule had a sustained HBV clearance and 7 eliminated HBeAg. The predictive response factors included the high ALT levels and the non-Asian ethnicity, but not the viral DNA pre-treatment levels. Most responses were generated during the second course of inoculations and most HBeAg clearances were preceded by a transient transaminase increase. This limited noncontrolled trial was followed by a controlled trial with a larger number of patients.

The new phase II trial was carried out in Indonesia. This study recruited 103 HBeAg positive chronic patients stratified based on the transaminase levels higher or lower than 1.5 upper normal limit (ULN) and randomized to receive four doses of the vaccine or placebo with monthly intervals.^15^ Eight months after the fourth immunization all the subjects received eight additional doses with monthly intervals. At the end of the first phase of four immunizations, it was observed a trend in favor of the vaccine toward a higher suppression of the viral replication, but this trend disappeared after the eight additional doses.^15^

In a European placebo controlled phase II trial, the patients received 8 monthly doses of vaccine or placebo. In this study, it was demonstrated that all the vaccinated subjects generated a significant anti-HBsAg lymphopro-liferative response but not to HBcAg. The T cell response was Th2 leaning, detecting IL-5 and IL-10 cytokines in the supernatants, but not IFN-y or IL-2. It was not observed any CD8+ T cell response by enzyme-linked immunospot (ELISPOT) in human leukocyte antigen (HLA) A2 patients.^16^

In vaccinated persons, the lympho-proliferative response increased during the first 5 months but it was stabilized or diminished afterward in spite of the continued vaccine administration.

In this study, three vaccinated patients and two from the control group showed a defined clinical response (normalization of transaminases and substantial reduction of the serum viral load). No correlation between the immunological and clinical responses was observed.

Meinyu Vaccine

The study was developed in 19 chronic HBV carriers with viral replication implying the monthly administration of six doses of the Meinyu anti-hepatitis B vaccine (Meiji Dairies Corp, Japan), based on the S variant of the HBsAg at a dose of 10 μg of the antigen by inoculation.

As a result of the study, it was detected a proliferative response in 31% of the vaccinated patients (4 out of 13 vaccinees). The seroconversion to HBeAg was 25%. Additionally, a strong production of IFN-y and TNF-a was detected in six patients together with a decrease of the viral DNA levels in the absence of cytotoxic T lymphocyte (CTL) activity.^17^ *Heberbiovac HB*

In a randomized, double blind, placebo controlled study designed to evaluate the therapeutic effect of the preparation of the HBV recombinant surface antigen in CHB, the Heberbiovac HB vaccine (HeberBiotec, Cuba) was administered five times by intramuscular route, in a doses of 40 μg and with a monthly frequency. The groups were equilibrated regarding the baseline and demographic variables.

The treatment was well tolerated and most patients had no adverse effects. At the end of the treatment, it was detected an important virological response regarding the frequency of patients having serum viral loads below 10^5^ copies per ml in the treated group with respect to the placebo group (47% *vs* 6%), in the same way, if the frequency of patients reducing their viral loads below 10^4^ copies per ml in both groups (26 *vs* 6%) is considered, the response continues to be important. In the treated group three patients completely cleared the virus, five patients developed anti-HBsAg antibodies-including the three patients that cleared the virus- and one of them became negative for HBsAg. The initially HBeAg positive responder cases became negative for this marker. The biochemical response had a high correlation with the virological response only in the case of the patients from the treated group. The histological response also was interesting from the clinical point of view since it was observed improvement in 30% as compared to the placebo group, existing correlation with respect to the virological response. Five cases of the treated group and none of the placebo group were considered responders in the global analysis since for them the virological (viral load below 10^4^ copies per ml), biochemical (transaminase normalization) and histological responses coincided. No differences were found regarding adverse events between the treatment and the placebo groups. In conclusion, the vaccine was considered safe and showed immunogenicity with an intermediate preliminary efficacy level.^18^

### Studies combining a Commercial Vaccine and Conventional Antiviral Therapies

The treatments that combine the therapeutic vaccination with conventional antiviral therapies have been used with the objective of favoring the development of an antiviral immune response. This strategy takes into consideration the finding related to the effect of lamivudine increasing the frequency of HBV specific T cells in peripheral blood as a result of the inhibition of the viral replication.^19^

The combined antiviral and vaccination therapy strategy must favor a better reactivity of the HBV T cell response, but also can be considered safer since it must avoid a large liver damage as a consequence of the immune system activation. However, there is no a single experience demonstrating that the vaccine activation of the specific immune response in CHB patients had provoked fulminant hepatitis B.

Engerix-B Experience on Low Viral Load Setting

In the year 2002, the results of a study evaluating the combined intradermal (ID) administration of Engerix-B vaccine (GlaxoSmithKline) with lamivudine were published.^20^ Nine CHB patients received six monthly administrations of the Engerix-B vaccine in combination with the daily administration of the antiviral lamivudine. Other group of six patients received the same treatment together with the daily subcutaneous administration of IL-2. Once the therapy ended, seven out of nine patients from the first group and two out of the five patients from the second had reduced their viral load to undetectable values. Four responders cleared the virus and had trans-aminase normalization; however, one of these patients showed a transient reactivation of the disease followed by spontaneous viral clearance 5 months afterward.

Regarding the immune response, it was found that the ID immunization was able to induce a low frequency of anti-HBsAg antibody producing B cells and helper T cells secreting predominantly IFN-y. The ELISPOT technique demonstrated the transient induction of cytotoxic specific T cells for HBV peptides in seven patients with the HLA-A2 haplotype. Summarizing, these preliminary results show that the HBsAg vaccine combined with antiviral or immune-modulator drugs can induce an antiviral response and as a consequence of this, clear the virus in patients with unfavorable prognostic.

Tokyo Mitsubishi Vaccine Experience on Low Viral Load Setting

In a clinical study designed to evaluate the combination of the therapeutic vaccination with the lamivudine therapy regarding safety, immunogenicity and preliminary efficacy, from a total of 72 CHB patients, 40 HBeAg positive patients and 32 with negative serology, all received daily 100 mg of lamivudine during one year, a total of 15 patients (9 HBeAg+ and 6 anti-HBeAg+) were additionally inoculated ID with the commercial vaccine containing HBsAg in alum.^21^

Twelve months after starting the therapy, all HBeAg(+) vaccinated patients had undetectable DNA levels, a proportion that was 15 out of 31 patients that only received lamivudine (48%). The HBeAg to anti-HBeAg seroconversion level was also significantly high (56% *vs* 16% in the lamivudine monotherapy group). An important element is that in the patients receiving the combined treatment there were no transaminase or viral DNA exacerbations, aspects that were observed in four out of 10 patients with monotherapy.

These studies evidenced that the combined therapy represent an effective therapeutic regimen, with few complications for CHB patients. However, it is interesting to point out that, in both cases, the ID immunization route was used, aspect that may be important for the vaccine functionality. As will be seen below, the same surface effective in a combined therapy with lamivudine, suggesting that the effect of the immunization route should not be missed and that if possibly represent an element of crucial importance for the future of this type of vaccination.

HBsAg from Engerix in an Adjuvant Formulation used under Viral Load Suppression

A study that closed the long clinical evaluation period of vaccine candidates in viral load suppression conditions is the report of Vandepapeliere et al.^22^ This study present the results of the clinical evaluation of one vaccine candidate based on an adjuvant adsorbed HBsAg formulation injected by IM route with a dose of 100 μg HBsAg, together with an oil adjuvant and potent immune-modulators, such as MPLA and QS21 saponin. The administration during 10 times starting from a reduced viral load did not show advantages regarding the virological response as compared to the control group, which was treated only with the antiviral.^22^

### Lessons on the Use of HBsAg-based Commercial Vaccines for Therapeutic Immunization

The accumulated knowledge with the therapeutic use of conventional vaccines and on the characteristics of the host immune response suggest that the immune-therapeutic strategies directed toward the induction of a strong and sustained T cell reactivity against HBV antigens are feasible and represent a hope for the satisfactory treatment of CHB patients.

The absence of immune stimulation against nuc-leocapsid antigens is probably a major immunological marker of the failure of the HBsAg-based therapeutic vaccination. Indeed, the goal of therapeutic vaccination in CHB is to trigger the same natural immune mechanisms that prevail during self-resolving acute hepatitis B or in seroconverting CHB. If an immune therapy fails to stimulate these immune responses, it will likely fail to induce seroconversion.

Formulation improvement in terms of antigen selection and vaccination strategy could be a way to overcome the previous mentioned difficulties. However, the envelope proteins certainly have characteristics, which justify their inclusion in a therapeutic vaccine. Indeed, envelope proteins contain numerous B- and T-cell epitopes^23,24^ and anti-envelope antibodies are thought to play a critical role in viral clearance by removing free viral particles from the circulation and by preventing reinfection of susceptible cells.^25,26^ On the contrary, massive amounts of HBsAg circulate in the serum of CHB patients, which could play a role in the maintenance of immune tolerance by T-cell exhaustion and by suppression of anti-HBs antibody production.^27^

In this line, HBcAg is probably a major candidate antigen to include in a therapeutic vaccine for CHB patients. The CD4+ and CD8+ T-cell cellular response to nucleocapsid epitopes are strongly enhanced and predominant during self-resolving acute hepatitis and during spontaneous or treatment-induced seroconversion of CHB.^28-30^

Despite none of the anti-hepatitis B commercial vaccines nowadays has demonstrated a sufficient level of clinical results to allow them to compete with the present treatments or that simply allow their introduction in the medical practice, these vaccines have created great expectations in the field of immunotherapy not only for the antihepatitis B immunotherapy setting but also fostering a dramatic development in other sceneries, such as HIV and cancer, among other chronic disease, transmissible or not. Hence, the status of this strategy demands the clinical evaluation of new immunotherapeutic concepts and the optimization of all related factors. In this sense, a vaccine candidate including HBcAg in addition to HBsAg is a realistic necessity in the development of such immune-therapeutic strategy.
